# Numerical Simulation Development and Computational Optimization for Directed Energy Deposition Additive Manufacturing Process

**DOI:** 10.3390/ma13112666

**Published:** 2020-06-11

**Authors:** Abhilash Kiran, Josef Hodek, Jaroslav Vavřík, Miroslav Urbánek, Jan Džugan

**Affiliations:** COMTES FHT a.s., Průmyslová 995, 334 41 Dobřany, Czech Republic; josef.hodek@comtesfht.cz (J.H.); jaroslav.vavrik@comtesfht.cz (J.V.); miroslav.urbanek@comtesfht.cz (M.U.); jan.dzugan@comtesfht.cz (J.D.)

**Keywords:** additive manufacturing, finite element method, directed energy deposition, residual stress, 316L steel

## Abstract

The rapid growth of Additive Manufacturing (AM) in the past decade has demonstrated a significant potential in cost-effective production with a superior quality product. A numerical simulation is a steep way to learn and improve the product quality, life cycle, and production cost. To cope with the growing AM field, researchers are exploring different techniques, methods, models to simulate the AM process efficiently. The goal is to develop a thermo-mechanical weld model for the Directed Energy Deposition (DED) process for 316L stainless steel at an efficient computational cost targeting to model large AM parts in residual stress calculation. To adapt the weld model to the DED simulation, single and multi-track thermal simulations were carried out. Numerical results were validated by the DED experiment. A good agreement was found between predicted temperature trends for numerical simulation and experimental results. A large number of weld tracks in the 3D solid AM parts make the finite element process simulation challenging in terms of computational time and large amounts of data management. The method of activating elements layer by layer and introducing heat in a cyclic manner called a thermal cycle heat input was applied. Thermal cycle heat input reduces the computational time considerably. The numerical results were compared to the experimental data for thermal and residual stress analyses. A lumping of layers strategy was implemented to reduce further computational time. The different number of lumping layers was analyzed to define the limit of lumping to retain accuracy in the residual stress calculation. The lumped layers residual stress calculation was validated by the contour cut method in the deposited sample. Thermal behavior and residual stress prediction for the different numbers of a lumped layer were examined and reported computational time reduction.

## 1. Introduction

Additive Manufacturing (AM) is a process of building a 3D structure by adding a material layer by layer. The process enables the construction of a complex structure and controlled microstructure. Directed Energy Deposition (DED) is one of the metal AM processes to fuse powder/wire material using focused thermal energy. The laser is used to create focused thermal energy that generates a melt pool. The powder is supplied at a controlled rate to the melt pool, and it solidifies as a laser head travels according to the scanning strategy. The moving laser source creates a track of deposited material. The scanning of the laser head over two dimensions forms a layer. Thus, adding a material layer by layer creates a 3D solid structure. A localized moving heat source and rapid cooling critically correlate with the part quality and structural integrity. Thermal energy distribution and duration of the thermal cycle have a direct effect on residual stress and distortion [1,2].

The experimental process and thermal gradient generated during the DED process have similarities with that of multi-track welding. Researchers have validated the welding model by in situ experimental results for residual stress and distortion [3], which has a close correlation to the DED system. Therefore, in the presented work a welding model was developed according to the requirement for a DED simulation.

Computational capability and improvement of numerical simulation tools enable the use of Finite Element (FE) modeling to predict thermal response [4,5,6,7,8,9,10,11,12] and estimate residual stress [13,14].

Numerous attempts have been made to develop the thermo-mechanical numerical model and experimental validation for the DED process using the weld model [15]. A limited resource was reported in optimizing computational time for the DED process considering a large number of layers in the printed sample. A large number of weld tracks and layers in additively manufactured parts was the challenge for the numerical simulation. The AM process simulation reported difficulties in computational time and large amounts of data management. There were several efforts to reduce computational time for the AM simulation. Ignoring phase changes, lumping of multiple welds was reported in the computational weld mechanics [16] to reduce simulation duration in the numerical approach. Inherent strain generated during welding was used to calculate the deformed shape inherited from the thermo-mechanical process [17]. The inherent strain method reduced the complex thermo-mechanical simulation to a sole mechanical that made the simulation much faster [14]. A spatial reduction method explores the possibility of reducing a 3D problem to a two-dimensional (2D) one [18,19]. However, the 2D simulation technique has limitations to analyze a large 3D structure. Again, these techniques in computational weld mechanics are interesting to simulate multiple welds for the AM process to reduce computational time.

A multi-weld simulation was computationally inefficient and not feasible for model preparation as it contained thousands/millions of weld seams in the AM component. The techniques in computational weld mechanics were combined systematically according to the DED method to reduce the computational time.

The objective of the presented work is to develop a welding numerical model to estimate residual stress at an efficient computational time for a DED printed large sample. Thermal simulation is crucial for the AM process simulation in the residual stress calculation. To prove the weld model can be used for DED simulation, thermal results from simulation compared with DED results. Therefore, the simulation of a single and multi-track deposition was carried out and validated with the experimentally measured thermal field.

At the first stage, thermal validation of the weld model for DED was carried out for a single-track deposition. The single-track deposition was extended to multi-track deposition to validate weld model consistency for AM simulation. A weld model for a single track was modified with a thermal cycle heat input to reduce a substantial amount of computational time. A significant amount of reduction in computational time enabled the development of a thermo-mechanical numerical model for a 3D solid structure that contained thousands of weld tracks.

At the second stage, the weld model with the thermal cycle heat input was simulated for an additive manufactured 3D solid structure. The thermo-mechanical model for a 35 mm size cube deposition was developed and optimized for computational time to simulate the complete process efficiently. The mechanical analysis was performed to predict the residual stress of the printed part. The predicted residual stress from the mechanical analysis was validated with the contour method results. Further Lumping layer method was coupled with the thermal cycle heat input to reduce thermo-mechanical simulation duration.

## 2. Materials and Methods

Material and its description in the numerical simulation will be introduced in the following chapter. Thermal behavior, thermo-mechanical behavior, and residual stresses of the printed part will also be described in detail.

### 2.1. Material

Austenite stainless steel 316L was used for the presented work. This material is of industrial interest because of its outstanding corrosion resistance, ductility, and high toughness. It attracts applications in different engineering fields. Additively manufacturing steel 316L extends its application for producing complex structures and expands its application to many sectors.

The temperature-dependent elastic-plastic material properties are imperative for a thermo-mechanical numerical calculation. A base plate made of 316L steel chemical composition was measured using an optical emission spectrometer (Bruker Q4 TASMAN, Bruker, Billerica, MA, USA). Austenitic stainless steel 316L (Sandvik Osprey LTD, Neath, UK) powder with a 50–150 µm particle size range was used. Chemical compositions of the powder and base plate material are listed in Table 1.

**Figure 1:**
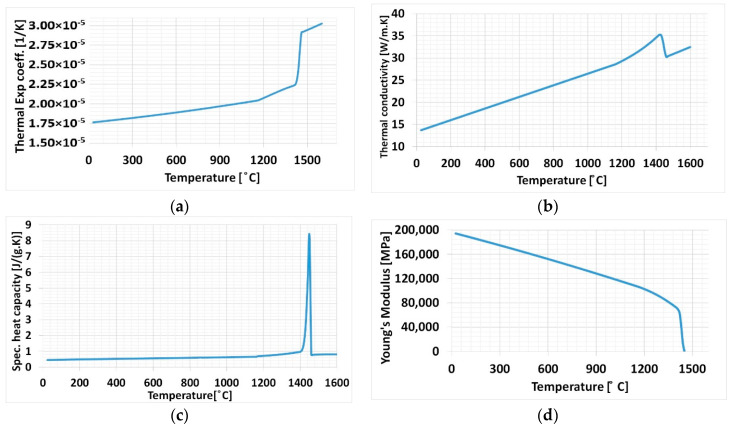
Temperature-dependent material properties for steel 316L (**a**) Thermal expansion coefficient; (**b**) Thermal conductivity; (**c**) Specific heat capacity; (**d**) Young’s Modulus

The chemical composition of steel 316L was used for JMatPro software (V11, Sente Software Ltd., Guildford, UK) to estimate temperature-dependent material properties shown in Figure 1.

Temperature-dependent material properties shown in Figure 1 were allotted to both baseplate and deposition material to all cases. Material properties variation can be observed in Figure 1a–c, and a steep rise can be noticed at the melting temperature of the material, these are similar to those found in the literature. The phenomenon is similar for Young’s Modulus behavior in Figure 1d.

### 2.2. Experimental Setup

**Figure 2:**
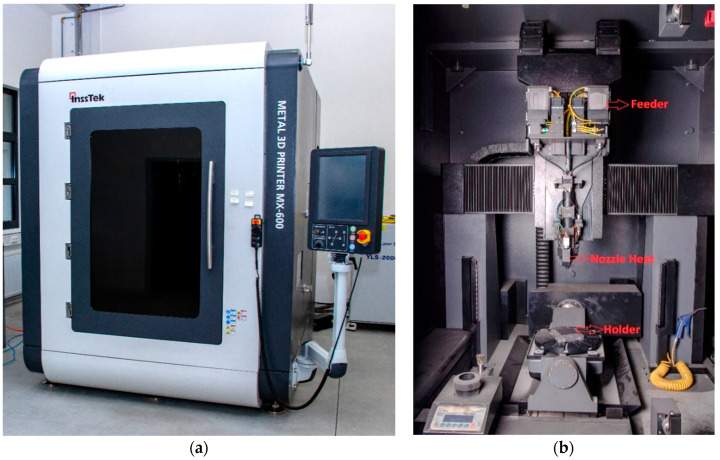
(**a**) InssTek MX-600 metallic deposition system (**b**) Deposition chamber.

The InssTek MX-600 metallic deposition system (InssTek, Daejeon, South Korea) with a 2 kW Ytterbium fiber laser equipped for the DED technique was used for the experiment. Figure 2a shows the InssTek MX-600 system. Figure 2b depicts the internal part of the deposition chamber. The powder was filled in the feeder mounted at the top. The deposition nozzle head contains a laser source allowing a multi-axis movement. Powder and argon gas flow coaxially to the laser path in the deposition nozzle head. A baseplate holder also enables a multi-axis movement and it is mounted at the bottom.

The laser beam diameter is 0.8 mm. The powder feeder is a gravity-based type with controlled gas pressure and a rotating wheel. The powder was fed at a constant feed rate of 3 g/min. Argon gas with constant pressure 2 bars was used as a shielding gas to protect the weld pool. The shielding gas consumption was 10 L/min and powder carrier gas 2.5 L/min. The distance between tracks was 0.5 mm (Hatching distance). The velocity of the laser head was 14 mm/s.

### 2.3. Thermo-Mechanical Model

Laser Metal AM DED process was analyzed using the numerical calculation in two steps. The first step predicted the thermal behavior of the model during the deposition process to estimate temperature variation. At the second stage, the output of thermal analysis results was used as a boundary condition to a mechanical analysis and residual stress was estimated in the AM component.

A coupled thermo-mechanical numerical model analysis was performed in the Abaqus version 2017 software (Dassault Systèmes, Vélizy-Villacoublay, France) using the welding model. An element birth technique was implemented in the thermo-mechanical AM process simulation [20,21]. According to the scanning strategy, elements/layers are activated at each time step. The temperature profile of the nodes from the thermal analysis was used as a thermal load in the mechanical analysis. The standard convection boundary condition is applied to the base plate and deposited material. A heat transfer coefficient for a different case is reported in the respective sections. A fixed displacement boundary condition was applied at the bottom edge of the base plate to replicate the experiment condition. A detailed explanation of the coupled thermo-mechanical procedure was reported in [22,23]. A numerical simulation was conducted in the computer powered by Intel i7 six-core processor (3.20 GHz) with 32 GB Random Access Memory (RAM).

#### 2.3.1. Thermal Modeling

The following formulas are used in commercial code Abaqus and numerical models will behave according to these basic physical laws. Heat distribution definition influences the temperature gradient from the weld zone to the base plate [4,24,25]. The deposition system employs a laser source for fusing material. Gaussian distribution laser energy for transient heat input analysis was used for the weld model.

The energy balancing equation is

(1) $$\underset{v}{\int }\rho U\;dV=\underset{s}{\int }q\;dS+\underset{v}{\int }r\;dV$$

where *V* is a volume of solid material, *S* is surface area, *ρ* is the density, *U* is the material time rate of the internal energy, *q* is the heat flux per unit area of the body, *r* is the heat supplied internally into the body per unit volume.

Heat conduction equation for the thermal field is given by [25]

(2) $$\frac{\partial }{\partial x}\left(k\left(T\right)\frac{\partial T}{\partial x}\right)+\frac{\partial }{\partial y}\left(k\left(T\right)\frac{\partial T}{\partial y}\right)+\frac{\partial }{\partial z}\left(k\left(T\right)\frac{\partial T}{\partial z}\right)+Q_{v}=\rho \left(T\right)C_{p}\left(T\right)\frac{\partial T}{\partial t}$$

where *k*(*T*) is the thermal conductivity, *ρ*(*T*) is the specific mass, *Cp*(*T*) is the specific heat. These quantities are the function of temperature. *Qv* is the volumetric heat flux.

The thermodynamic boundary condition consists of heat transfer through convection and radiation on the external surface of the sample.

Newton’s law computes heat flow through gas or liquid:

*q_c_* = *Ah_c_*(*T* − *T*_0_) (3)

where *q_c_* is convective heat flow, *h_c_* heat transfer coefficient, *A* heat transfer area, *T* is the temperature on the surface of the material, *T*_0_ is the temperature of the surrounding environment i.e., deposition chamber.

Radiation heat flux governed by the Stefan-Boltzmann law:

*q_r_* = *ε_r_σ_r_*(*T*^4^ − *T*_0_^4^) (4)

where *q_r_* radiant heat, *ε_r_* is the emissivity (0.9), *σr* is the Stefan-Boltzmann constant (5.67 × 10^−8^ W/m·K).

#### 2.3.2. Mechanical Modeling

The mechanical computation is the second stage of the calculation. The resolution of the mechanical equation is based on the equation of static equilibrium [26].

(▽ · *σ* + *F*) = 0 (5)

where *σ* is the stress tensor, *F* is the body force and ▽ is the divergence operator. The global deformation during the mechanical analysis of welding was defined as the sum of the deformations (elastic strain *ɛ^e^*, plastic strain *ɛ^p^*, thermal strain *ɛ^th^*) [27].

*ε^total^* = *ε^e^* + *ε^p^* + *ε^th^* (6)

Mechanical constitutive law is stated as

*σ* = *C*:*ε^e^* (7)

where *C* is the fourth-order elastic stiffness tensor. A phase transformation calculation was not considered for stable austenitic steel 316L during the thermo-mechanical analysis.

### 2.4. In Situ Temperature Measurement

The temperature course during the deposition process was recorded using a thermocouple. The thermocouples were welded on the base plate to record temperature variation during the laser head movement. The position of the thermocouple was defined for each case in the respective section. Several thermocouples were welded on the base plate in each case and a thermocouple with consistent data was selected for comparison with numerical results. Thermocouple results considered for thermal validation during the different cases are shown in the respective section.

**Figure 3:**
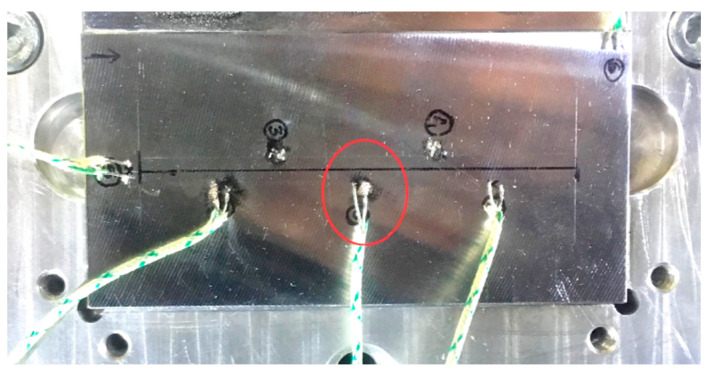
Thermocouples welded on the base plate.

The temperature course on the base plate was recorded using a thermocouple (type “K”) recorded at the interval of 1 s. Figure 3 shows the example of thermocouples welded on the base plate for a single-track deposition case. Data from a thermocouple located at the center (marked in a red circle) was used for comparison to numerical data for a single-track deposition validation. Thermocouple considered for other cases is shown in respective sections.

### 2.5. Residual Stress Evaluation

**Figure 4:**
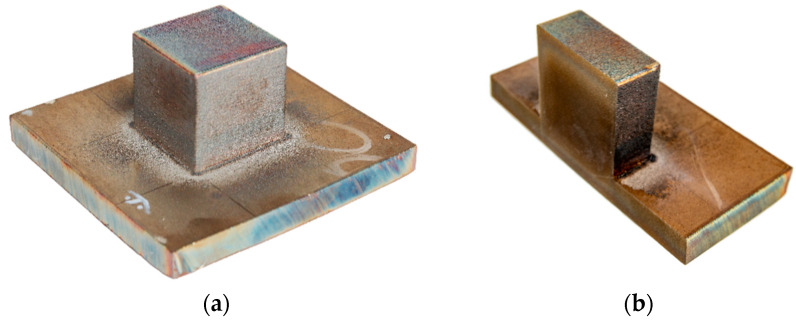
(**a**) Printed sample (**b**) Cut sample for Contour method measurement.

The contour method is an indirect technique to map 2D residual stress based on elastic stress relaxation [28,29]. A printed 3D cubic sample was cut at the plane inside the sample where the residual stress was compared with the numerical result. Figure 4a shows a 35 mm cubic sample considered for the residual stress calculation using the contour method. A cubic sample was cut using a wire electric discharge machine (diameter of the wire 0.25 mm). Figure 4b shows the sample cut in the middle to map residual stress perpendicularly to the plane of the cut. The surface geometry of the cut plane was scanned using Optical Precision Measuring Machine (OPMM) ATHOS Capsule (Carl Zeiss AG, Oberkochen, Germany). The sliced plane coordinate was implemented as a boundary condition for the FE analysis software. Planar residual stress was calculated in Finite Element Analysis (FEA) software with 8-point isoparametric elements, characteristic element length was 1 mm and Young’s modulus (*E*) = 192.968 GPa and Poisson’s ratio (*ν*) = 0.2989.

The advantage of the contour method has no limit on the size of the structure to analyze the residual stress, unlike the X-ray or neutron diffraction method. The contour method is cost-effective and carried with simple accessible tools. Residual stress from the numerical calculation for the cubic 3D structure was validated to experimentally determined using the contour method.

## 3. Results

The described models and methods were used for simulation of a single track and multi-track depositions with different numerical approaches. Details are described in the following subchapters and it is essential to compare the speed and accuracy of the calculations at the end of the article. Determination of the residual stress in the 3D metallic printed component is necessary for this process and its parameters. Heat input and thermal boundary condition calibration are crucial, a slight deviation in predicting temperature evolution directly influences the calculation of residual stress during the thermo-mechanical simulation. Therefore, the thermal weld model was developed for a single track and multi-track deposition. The developed thermo-mechanical model can be used for a generally 3D structure deposited by the DED AM process. The numerical results were confronted with the experimental results.

### 3.1. Single-Track Deposition

**Figure 5:**
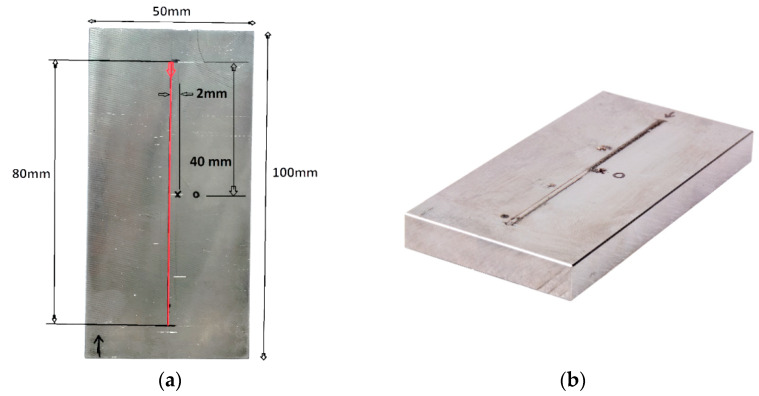
(**a**) Weld track and specimen specification (**b**) single-track deposited sample.

The welding model thermal result was verified by the DED process for a single-track length of 80 mm. The base plate was 10 mm thick. Deposition track, thermocouple position (cross marked) and the dimension of the base plate are shown in Figure 5a. A redline and direction in the center of the base plate indicate the path and direction of the laser head movement. Deposited material was projected above the surface of the base plate in the defined path as could be noticed in Figure 5b.

**Figure 6:**
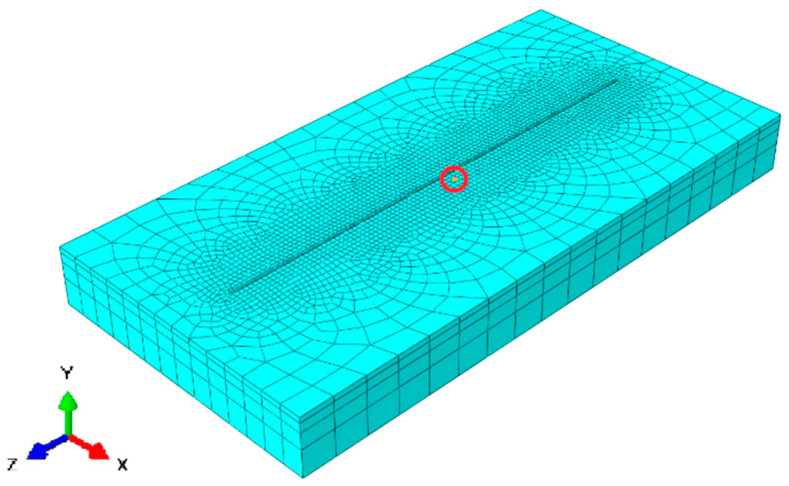
The meshed model with marked nodal position.

Figure 6 shows the 3D finite element mesh model with fine mesh around the deposition zone. Mesh gradually coarsened in the base plate away from the deposition zone where the thermal gradient is lower. Hexahedral elements with heat transfer DC3D8 (8-node linear transfer brick) from the Abaqus library was used for the thermal FE analysis. A mesh sensitivity analysis was performed to generate results independent of mesh structure and element size. The course elements away from the deposition zone and directional mesh along with the thickness of the base plate were created to minimize the number of elements for computation time optimization. An FE model consisted 11,930 hexahedral heat transfer elements. The temperature of 25 °C was assigned to the base plate at the initiation of numerical calculation. The heat transfer coefficient for the single-track simulation was 25 W/(m²·K). A transient heat input method was implemented for a single-track simulation. The node stated inside the red circle (Figure 6) is the position where the nodal temperature was extracted to be compared to thermocouple results from the experiment. The nodal position and thermocouple position was selected at the same location for the precise comparison of results.

**Figure 7:**
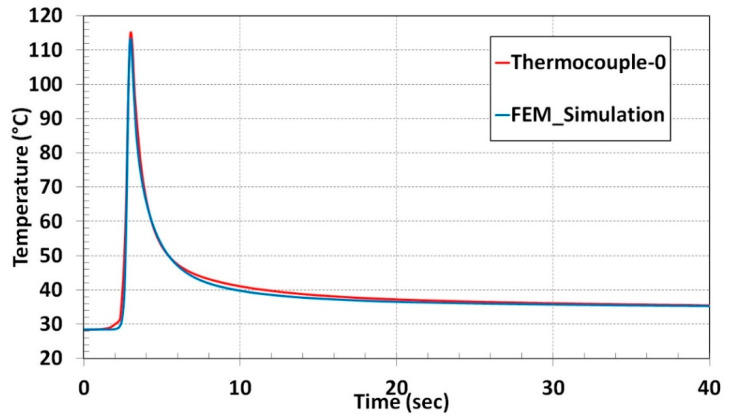
Numerical result and experiment temperature comparison for single-track deposition.

Temperature calibration results are depicted in Figure 7. The red curve was derived from thermocouple data. Steep temperature rise was recorded when the laser head was approaching the thermocouple, the maximum temperature of 113 °C was reached when the laser head was near the thermocouple. The temperature fall in the graph is a consequence when the laser moved away from the thermocouple position.

The blue curve of the nodal temperature was derived from the node that is marked inside the red circle shown in Figure 6. Temperature vs. Time graph for the thermocouple-0 and nodal temperature shows excellent agreement depicted in Figure 7.

### 3.2. Multi-Track Deposition

**Figure 8:**
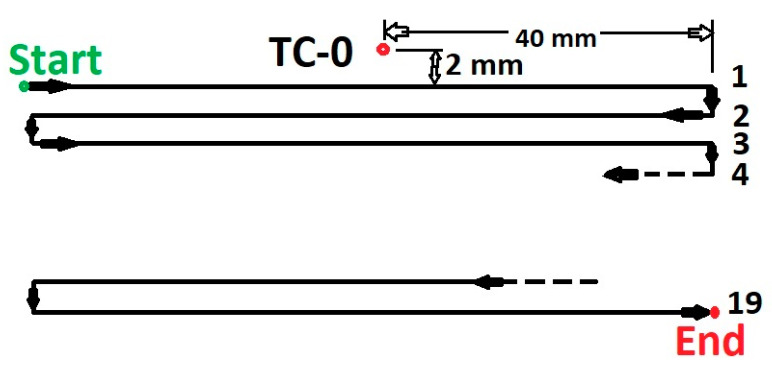
Scanning strategy and thermocouple position for multi-track deposition.

**Figure 9:**
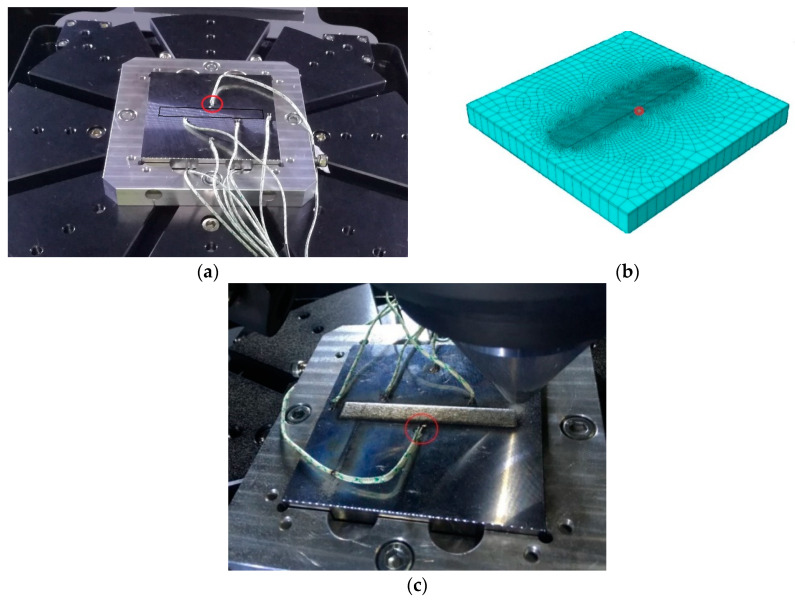
(**a**) Picture of 19 tracks deposition base plate mounted on 3D printing holder and thermocouple position marked with red circle selected to compare temperature evolution with numerical results; (**b**) Meshed model and the nodal position selected for comparison with the experimental results; (**c**) Deposited sample captured after the experiment.

A single-track numerical model is extended to a multi-track investigation with 19 tracks. The multi-track analysis is to check the consistency to adopt for an AM simulation and accuracy. 316L steel base plate of thickness 10 mm, 96-mm² cross-section area was used for the deposition of multi-tracks. The scanning strategy for 19 tracks is shown in Figure 8. The meshed model is displayed in Figure 9b contains 16,266 hexahedral heat transfer elements. Elements along the thickness of the base plate were reduced to two elements. Fine elements at the surface of the base plate were retained to ensure the desired accuracy and to minimize computational time. A high density of elements in and around the deposition zone was generated to keep a high resolution for a large temperature gradient.

The temperature evolution during deposition was recorded from the thermocouple (marked as TC-0) and the position of the thermocouple is shown in Figure 8.

The 19 tracks deposition area is marked in the dark rectangular box shown in Figure 9a. The thermocouple position marked by the red circle illustrated in Figure 9a,c was selected for comparison to the nodal temperature evolution from the simulation. The nodal temperature was derived from the node marked inside the red circle in the meshed model shown in Figure 9b.

**Figure 10:**
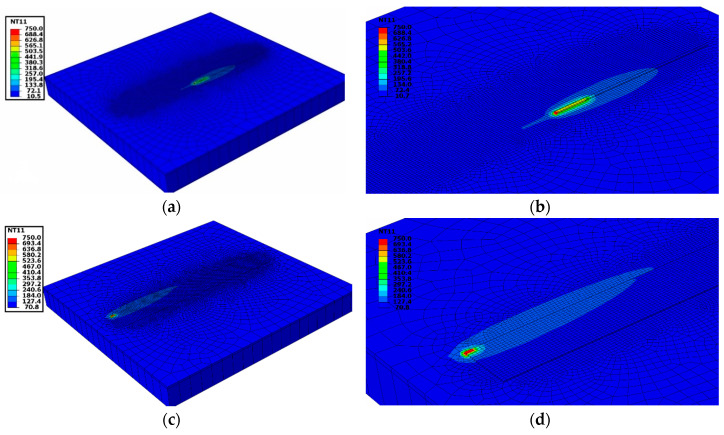
(**a**) Temperature profile during 1st track deposition; (**b**) Detail view of 1st track deposition; (**c**) Temperature profile during 19th track deposition; (**d**) Detailed view at the end of the deposition.

Figure 10 shows the temperature contour during the 19th track deposition. Figure 10a shows at the end of a welding step in the first track near the node consider for temperature comparison. Figure 10b is the enlarged image and shows the temperature gradient around the deposition area. Activated elements can be noticed behind the current welding element shown in Figure 10b. Inactive elements for remaining tracks are not visible.

Figure 10c shows deposition at the end of the 19th track. The detailed view in Figure 10d shows the temperature gradient in the printed track and base plate. All activated elements representing 19 tracks are visible. Elements in the printed track allow a heat flow from succeeding deposition and are involved in thermal interaction until the end of the cooling step.

**Figure 11:**
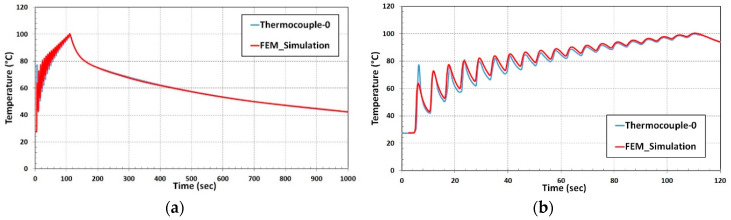
(**a**) Thermal validation for multi-track deposition; (**b**) Enlarged graph of Temperature vs. time during 19 track deposition.

A good agreement of temperature evolution recorded during the deposition and cooling process is shown in Figure 11a. The enlarged graph of temperature variation during the deposition of 19 tracks showing the close agreement of numerical results with thermocouple data is depicted in Figure 11b.

### 3.3. Thermal Cycle for Single-Track Deposition

The concept of a ‘thermal cycle’ heat input is to reduce considerable computational time compared to the transient heat input numerical simulation for the same amount of a mesh element, boundary condition, and other process parameters. The transient heat input method thermal simulation consumed enormous computational time during a single track (around 2 h) and for 19 tracks (around 7 h) simulation. A solid cube of dimension 35 mm contains nearly 10 thousand tracks and 140 layers. Transient heat input numerical calculation takes tremendous computation time to compute such a 3D structure for thermo-mechanical simulation.

During the thermal cycle heat input for single-track deposition, complete deposition track was considered to be one block and heat was introduced based on balancing total energy. Integrating multiple steps during the transient heat input numerical calculation into a single step in the thermal cycle reduces the considerable computational time.

**Figure 12:**
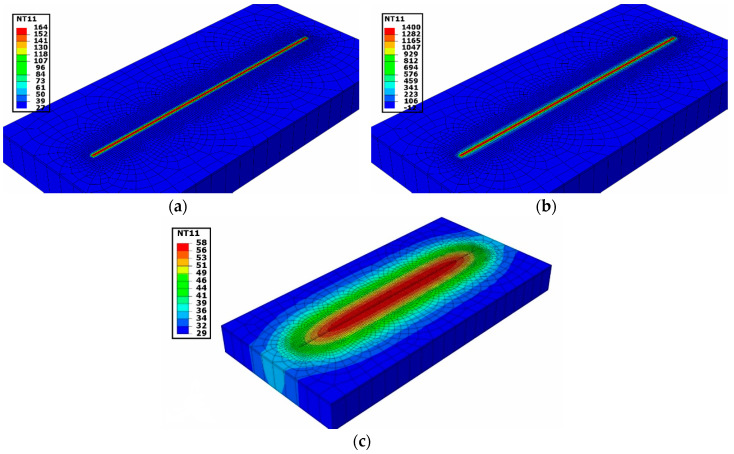
Temperature evolving during thermal cycle heat input method for single-track simulation. (**a**) 2.358173 s, (**b**) 2.6155 s and (**c**) 5.7 s

Figure 12 shows the temperature field for the thermal cycle heat input. Elements representing deposition material of length 80 mm were considered to be a single block. The nodal temperature was evolved concurrently in all elements, which represent the deposited material during DED shown in Figure 12a. The temperature of 164 °C uniformly was developed over the deposition elements at the beginning of the simulation (step time = 2.358173 s). A maximum temperature of 1400 °C in the course of heat input at 2.6155 s time step was reached as shown in Figure 12b. Figure 12c represents the cooling step at 5.7 s where the temperature drops to 58 °C in the deposited elements. The gradient of temperature around the deposition zone represented with different colors is depicted in Figure 12c.

**Figure 13:**
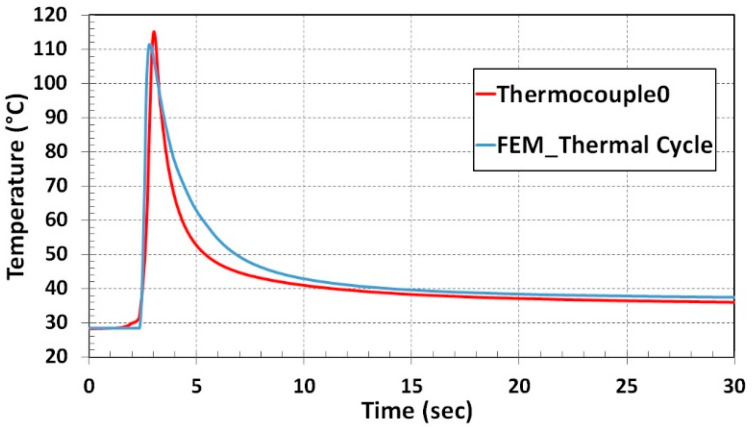
Numerical result and experiment temperature comparison for thermal cycle heat input method.

The nodal temperature variation during the single-track deposition process was compared to thermocouple results from the experiment. The computational time during the thermal cycle heat input drops to 5% of transient heat input computational duration for the single track. A temperature variation profile from thermal cycle heat input numerical results comparison is illustrated in Figure 13. A marginal deviation during the temperature drop phase was noticed in the numerical prediction. Apart from that, the nodal temperature from the numerical result shows a good agreement with the thermocouple curve.

### 3.4. Thermal Cycle Heat Input Method for 3D Structure

**Figure 14:**
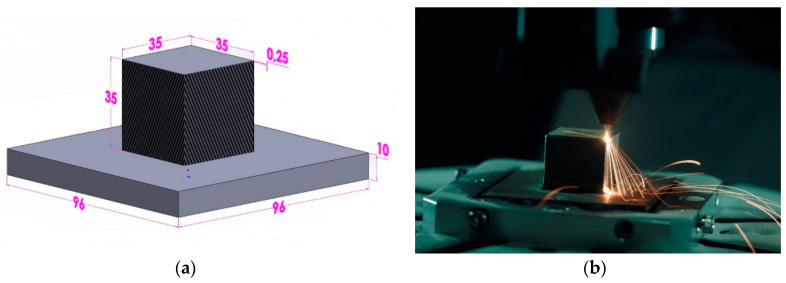
(**a**) A CAD model of the cubic structure with dimensions in millimeters; (**b**) 3D cubic structure deposition.

The validated thermal weld model with single and multi-track DED was implemented to the 3D metallic structure for residual stress calculation. A simple cubic 3D structure was considered to be implemented the thermo-mechanical weld model to test different computational time optimizing FEM techniques. A Computer-aided design (CAD) model for FEM simulation contains 140 thin square blocks that represent layers in the deposited sample of 0.25 mm thickness that was created using SolidWorks software (V2018, Dassault Systèmes, Vélizy-Villacoublay, France). The dimension of the structure is shown in Figure 14a. Thermal and mechanical temperature-dependent material properties used from the graph generated from JMatPro software are shown in Figure 1.

The DED deposition process of a cube of 35 mm with a process parameter was reported in the experimental Section 2.1. A Contour filling scanning strategy was employed and around 10,000 weld tracks were deposited. The deposition lasted 8 h. Figure 14b shows the deposition of the cube structure captured during the deposition of topmost layer of the cube.

The thermal cycle for the single-track concept has extended to a 3D structure simulation. Similar to the thermal cycle heat input for a single track, here the complete layer was considered to be one block.

The thermal cycle heat input was based on the principle of balancing total energy. The energy input was balanced by considering the duration and volume to which heat flux was introduced. Heat flux duration was limited based on the following two factors:

- Heat flux allowed for a fraction of the total time required for layer deposition.
- The intensity of the heat flux is set according to the melting point of the material.

**Figure 15:**
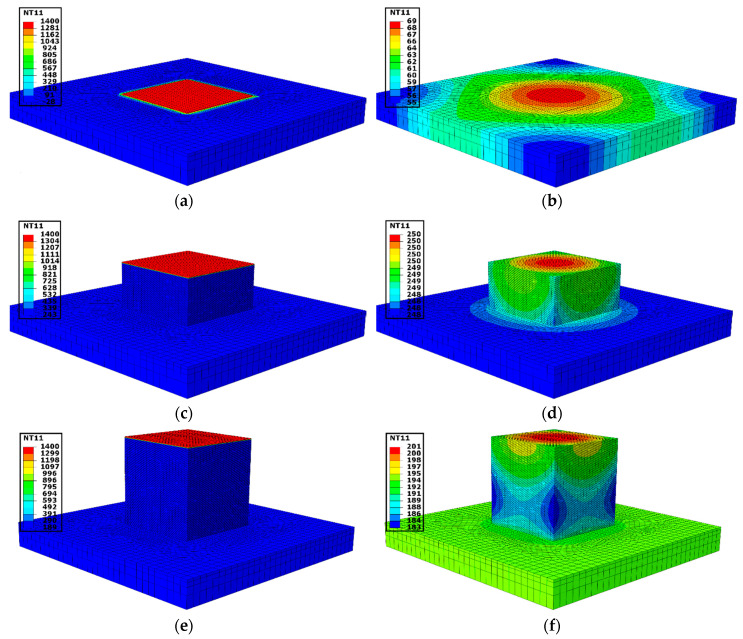
Temperature (°C) evolution at different layers during thermal simulation of 3D cubic structure. The nodal temperature where the first layer (**a**), the 70th layer (**c**) and the 140th layer (**e**) of element reaches 1400 °C; and temperature gradient at the end of the step time for the first layer (**b**), the 70th layer (**d**) and the 140th layer (**f**).

During the thermal cycle heat input, elements of the targeted layer were activated, and the heat was introduced for activated elements simultaneously. The remaining upper layers are in the inactive state. Figure 15a shows the nodal temperature where the first layer of element reaches 1400 °C. Activated layer elements interact with lower layer/base plate elements. Therefore, at the end of the first layer temperature gradient is noticed on the base plate in Figure 15b. Elements were activated for the duration of the time required for one-layer deposition during the in situ experiment. A succeeding layer (Upper layer) was activated on completion of the current layer deposition duration. Similarly, the development of the 70th layer is shown in Figure 15c when the nodal temperature of the layer is at 1400 °C. The gradient of the temperature on the deposited layer and the base plate is depicted in Figure 15d. Figure 15e shows all 140 layers of activated elements. At the end of the step time for the 140th layer temperature gradient on the deposited layer and the base plate is clearly shown in Figure 15f. The heat was introduced layer by layer in a “cyclic manner” for 140 layers.

#### 3.4.1. Thermal Validation for 3D Structure Simulation

**Figure 16:**
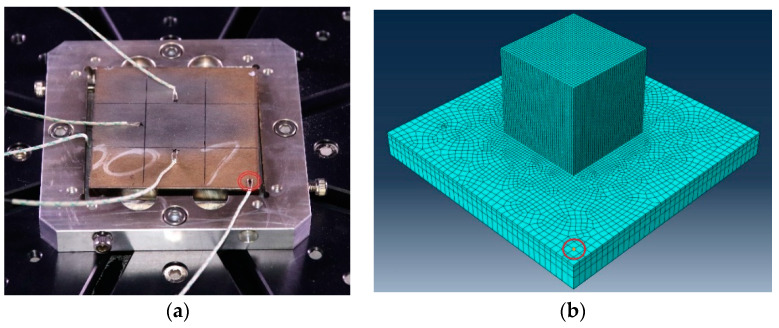
(**a**) Picture captured before the experiment illustrates the thermocouple position; (**b**) Meshed model marked with the nodal position.

Temperature variation during deposition and cooling down to room temperature was recorded from the thermocouple. The thermocouple marked by the red circle was considered for the comparison to numerical results. It is located at 35.35 mm diagonally from the cube nearest edge shown in Figure 16a. The nodal temperature from the node was marked inside the red circle in the meshed model compared to the experiment results. A heat transfer DC3D8 element was selected from the Abaqus library for thermal analysis. The number of elements was limited between 60 to 75 thousand for the thermal cycle model and four cases in the lumping layer model (discussed in the next Section 3.4.2). A narrow difference in the number of elements was maintained for all cases to ensure results are independent of the mesh structure. One element along with the thickness of the layer was generated for all five cases. The heat transfer coefficient of 8 W/m²·K was used for a 3D cubic structure thermal analysis. The initial temperature of 25 °C was used for the simulation.

**Figure 17:**
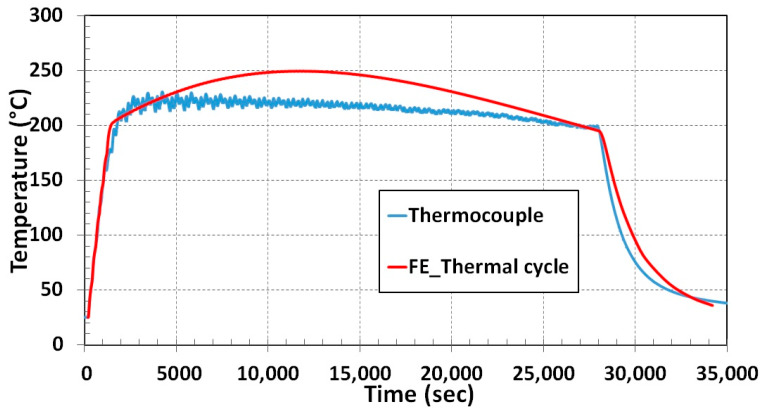
Temperature comparison between experiment and simulation for 3D structure.

Temperature variation during the deposition process for 8 h and cooling down to room temperature for nearly 3 h was recorded by a thermocouple. The blue curve in Figure 17 represents temperature fluctuation during the in situ experiment. Temperature fluctuation prediction from the numerical simulation shows the same trend of thermocouple results depicted in Figure 17. Around 25 °C deviations of temperature in the middle of deposition were noticed in numerical results from thermocouple data. The possible reason for difference in temperature prediction during the process is method of heat introduction (thermal cycle heat input) to a layer simultaneously.

#### 3.4.2. Residual Stress Validation

**Figure 18:**
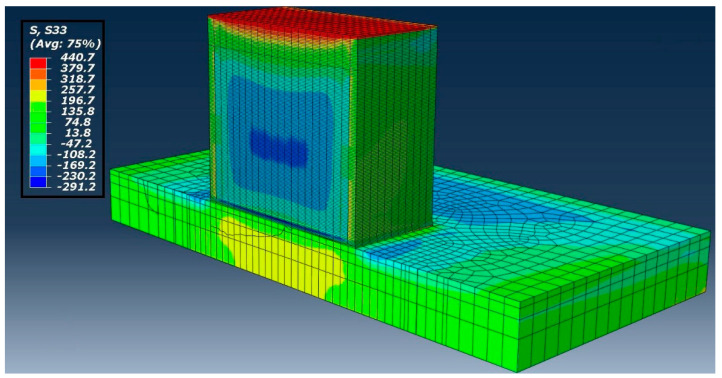
Residual stress of the Z component in the sliced plane.

The thermal model with the same mesh and element order was considered for mechanical simulation with a 3D stress element. A temperature profile from the thermal analysis was used to drive a mechanical analysis. The residual stress of the AM component calculation includes the model cooling rate to room temperature. Von Mises stress perpendicular to the sliced plane (X-Y plane) i.e., in the z-direction mentioned as S33 is shown in Figure 18 compared to contour results.

**Figure 19:**
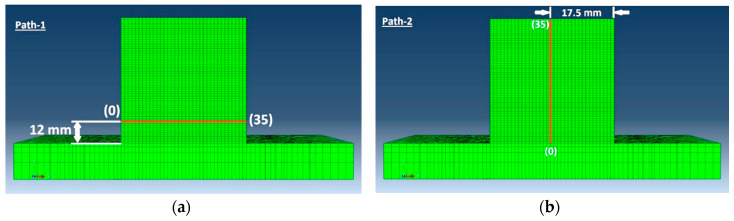
(**a**) Path-1 position and direction start from the left edge of the cube marked as (0), ends at the right edge (35); (**b**) Path-2 position and direction start from the surface of the base plate marked as (0) ends on the surface of the printed cube i.e., 35 mm height marked as (35).

Two paths were considered in the sliced plane as shown in Figure 19. Path-1 was parallel to the base plate plane marked in Figure 19a and Path-2 was perpendicular to the base plate surface marked in Figure 19b.

**Figure 20:**
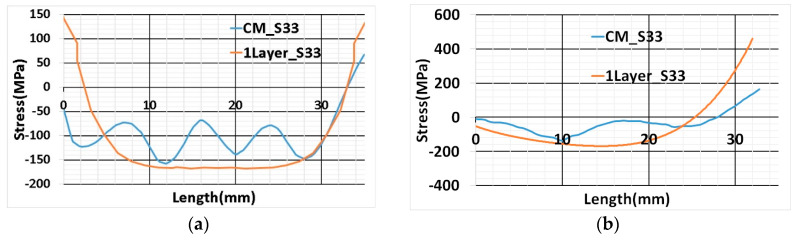
(**a**) Path-1 position and direction start from the left edge of the cube marked as (0), ends at the right edge (35); (**b**) Path-2 position and direction start from the surface of the base plate marked as (0) ends on the surface of the printed cube, i.e., 35mm height marked as (35).

The blue curve (CM_S33) in Figure 20a,b represents a contour cut method result. Wavy nature of the stress inside the additively manufactured sample was determined from the contour cut method shown in Figure 20a. The possible reason is the influence of multi-track transient heat input and scanning strategy. The scanning strategy for cube deposition was contour filling and for every layer deposition, the starting point shifted by 90 degrees. However, in numerical results, uniform stress was predicted inside the sample. It is the consequence of elements activation in a layer simultaneously. Simulated results from thermo-mechanical modeling have a good agreement with contour method results. Path-1 results revealed stress estimation inside the sample has a close agreement with the contour method curve shown in Figure 20a Path-1 numerical results show symmetry of stress variation. This behavior was expected because of the thermal cycle method of heat input.

Path-2 numerical results (1Layer_S33) show a very close agreement with the contour method result and the trend of stress variation is similar as shown in Figure 20b. The difference in the result can be noticed at one of the edges in two paths (At the left edge of the graph in Path-1 and right edge in the Path-2) depicted in Figure 20a. The possible reason could be residual stress at the edges of the sample from the contour method is inaccurate. Edge results are prone to error; cutting at the edge influence residual stress relaxation [30].

### 3.5. Lumping of Layers for 3D Cubic Structure

The lumping of layers method complies with the thermal cycle technique to reduce computational time further. The lumping of layers was implemented by merging multiple layers into a single layer in a numerical model. Depending on the number of layers lumping, the thickness of a layer in the CAD model increased. For example, in the case of two-layers lumping thickness in the CAD model of each layer doubled and the total number of layers drops to half (140 layers reduces to 70 layers in the cubic model case considered in the presented work).

To understand the lumping of layers effect on the results and to determine the limit of lumping, four cases are considered.

- Two-layers lumping
- Four-layers lumping
- Seven-layers lumping
- Ten-layers lumping

Total energy input was balanced for different lumped layers by restricting the duration of the heat input.

#### 3.5.1. Thermal Validation

**Figure 21:**
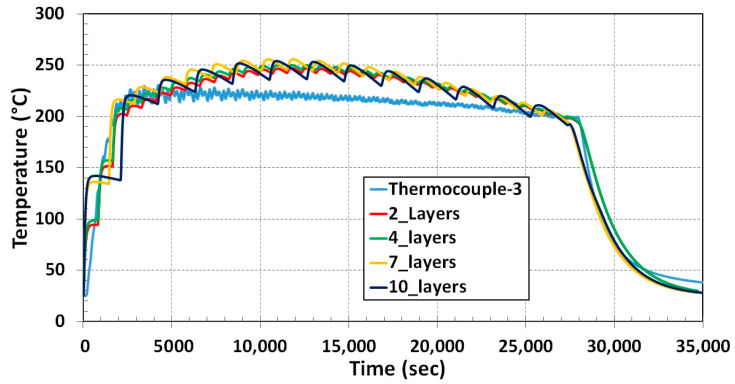
Temperature comparison for all four cases of lumping layer with the experiment result.

Thermal simulation results for all four cases show the same tendency of temperature variation compared to the single layer in the previous section. As the number of lumping layers increases, the fluctuation of temperature for each layer increases. Ten-layers lumping temperature fluctuation is large compared to two-layers lumping during the deposition period depicted in Figure 21. This is due to prolonged step time for high lumping numbers. The position of the thermocouple and node is shown in Figure 16.

#### 3.5.2. Residual Stress Validation

**Figure 22:**
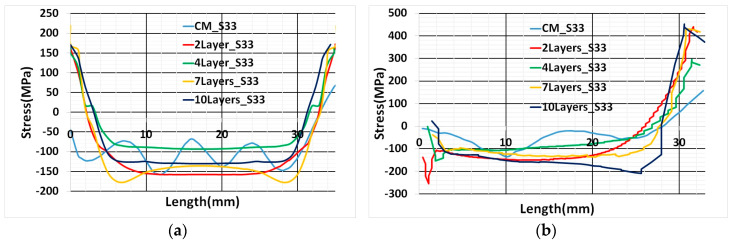
(**a**) Residual stress in the Path-1 comparison with contour method; (**b**) Residual stress comparison in the Path-2.

Numerical results of residual stress for all four cases show a good agreement with the contour method along Path-1. A slight deviation from the contour method results grows as the number of lumping layers increases. This is evident for residual stress estimation inside the sample for seven- and ten-layers lumping shown in Figure 22a. Seven and ten-layers of lumping decrease computational time, but numerical results initiate to diverge from contour results.

Path-2 numerical results also show good agreement similar to thermal cycle heat input results and a slight deviation for ten-layers lumping from other cases depicted in Figure 22b.

### 3.6. Computational Time

**Figure 23:**
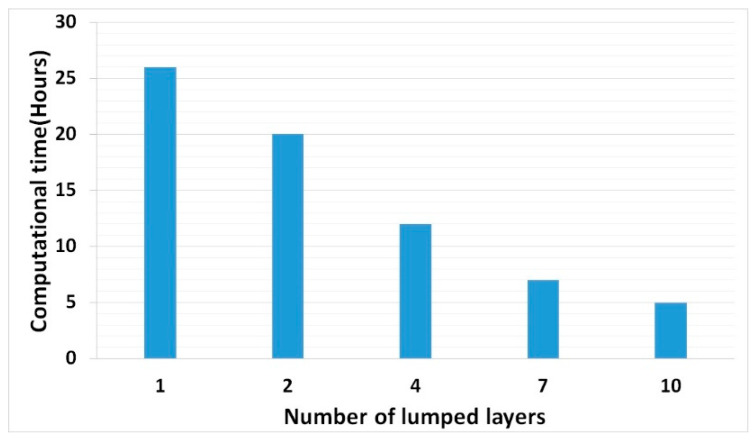
Thermo-mechanical simulation duration for different lumped layers for the cubic sample.

The estimated computational time for a 3D solid structure thermo-mechanical simulation is several weeks with the thermally validated weld model for single and multi-track. (Computational time dropped down to 26 h by implementing the thermal cycle for single layer). Further layers lumping reduced simulation time for both thermal and mechanical analyses linearly as shown in Figure 23.

The 3D structure considered for implementing the thermal cycle and lumping layer is a simple symmetric structure. Therefore, seven and ten layers of lumping case residual stress calculation retained accuracy. Two and four layers of lumping residual stress calculations are more precise and suitable for implementing a complicated structure.

## 4. Conclusions

The goal of this work was to develop a weld model for DED simulation and implementing different FEM techniques to evaluate the computational time. That means developing the FE model targeting an AM simulation for the object size of the centimeter or meter scale range. Thermal validation has proved the weld model is compatible with the DED thermo-mechanical simulation. Other main results of this work are described in the following points

- The validated weld model for the single and multi-track simulation with the transient heat input has limitations in terms of the computational time. The transient heat input model is not reasonable for an additively manufactured solid structure numerical simulation.
- The thermal cycle heat input method reduces both job preparation effort and computational cost. Drastic reduction with simulation time prompt to implement a 3D solid AM structure.
- The limitation of the thermal cycle heat input is ignoring the scanning strategy and local anisotropy. The work intends to do a macroscopic analysis and calculates the residual stress at a reasonable time but not a microscopic analysis of the scanning strategy effect in the AM sample.
- The thermal cycle heat input method was successful to reduce computational time for a 3D solid structure with a good agreement of thermal and residual stress results from the experiment. Moreover, further lumping of layers linearly reduced the simulation time.

These results demonstrate the combined importance of the thermal cycle and lumping layer to evolving challenges in the simulation of large additively manufactured engineering parts. It provides a modeling framework for an AM simulation to predict residual stress before manufacturing without volume/dimension limit and take measures to improve part quality. This could enhance the mechanical properties and life cycle of the additively manufactured parts. This model provides multiple options to adopt the model depends on the size of the structure and accuracy required. Reduction in a computational cost lowers the price of manufacturing at different stages and helps in manufacturing without interrupting the supply chain.

## Figures and Tables

**Table 1:** Powder and base plate Steel 316L chemical composition (wt. %).

	Fe	Cr	Ni	Mo	Mn	Si
Powder (316L)	Bal.	17.2	10.4	2.3	1.3	0.8
Base Plate (316L)	Bal.	16.24	10.49	2.14	1.12	0.44
